# Antimicrobial Resistance in *Escherichia coli* Isolates from Healthy Food Animals in South Korea, 2010–2020

**DOI:** 10.3390/microorganisms10030524

**Published:** 2022-02-28

**Authors:** Hyun-Ju Song, Su-Jeong Kim, Dong Chan Moon, Abraham Fikru Mechesso, Ji-Hyun Choi, Hee Young Kang, Naila Boby, Soon-Seek Yoon, Suk-Kyung Lim

**Affiliations:** 1 Bacterial Disease Division, Animal and Plant Quarantine Agency, 177 Hyeksin 8-ro, Gimcheon-si 39660, Gyeongsangbuk-do, Korea; shj0211@korea.kr (H.-J.S.); kimsujeong27@korea.kr (S.-J.K.); ansehdcks@korea.kr (D.C.M.); wlgus01@korea.kr (J.-H.C.); kanghy7734@korea.kr (H.Y.K.); nailaboby@korea.kr (N.B.); yoonss24@korea.kr (S.-S.Y.); 2 Division of Antimicrobial Resistance, National Institute of Health, Osong Health Technology Administration Complex, 187, Osong eup, Cheongju-si 28159, Chungcheongbuk-do, Korea; 3 Department of Pathology and Microbiology, College of Medicine, University of Nebraska Medical Center, Omaha, NE 68198-5900, USA; amechesso@unmc.edu

**Keywords:** antimicrobial resistance, *E. coli*, food animals

## Abstract

Antimicrobial-resistant bacteria in food animals pose a major public health threat worldwide. In this study, we aimed to assess the antimicrobial resistance profiles and resistance trends of commensal *Escherichia coli* isolated from the feces of healthy cattle, pigs, and chickens in South Korea during 2010 and 2020. A total of 7237 *E. coli* isolates (2733 cattle, 2542 pig, and 1962 chicken isolates) were tested for susceptibility towards 12 antimicrobials. About 48%, 90%, and 97% of cattle, pig, and chicken isolates, respectively, were resistant to one or more antimicrobial agents. Cattle isolates presented low resistance (<15%) to most of the tested antimicrobials. In contrast, chicken and pig isolates demonstrated a relatively high (>45%) resistance rate to ampicillin, chloramphenicol, streptomycin, and tetracycline. We observed high ciprofloxacin and nalidixic acid resistance rates in chicken (76.1% and 88.6%, respectively), isolates in pig (12.7% and 26.7%, respectively) and cattle (2.7% and 8.2%, respectively) isolates. Notably, a very small proportion of isolates (<5%) from cattle, chickens, and pigs demonstrated resistance to amoxicillin/clavulanic acid, cefoxitin, and colistin. We identified ceftiofur resistance in a small proportion of chicken (8.8%), pig (3.7%), and cattle (0.7%) isolates. We noted an increasing but fluctuating trend of ampicillin, amoxicillin/clavulanic acid, ceftiofur, cefoxitin, chloramphenicol, ciprofloxacin, and streptomycin resistance in pig isolates. Similarly, the ampicillin, ceftiofur, and chloramphenicol resistance rates were increased but fluctuated through time in chicken isolates. Overall, 56% of the isolates showed multidrug-resistant (MDR). The proportion of MDR isolates was low in cattle (17.1%); however, this proportion was high in chickens (87.1%) and pigs (73.7%). Most of the resistance patterns included streptomycin and tetracycline in pigs and cattle, and ciprofloxacin and nalidixic acid in chickens. In conclusion, this study showed high resistance of commensal *E. coli* isolated from major food animals in Korea to commonly used antimicrobials including critically important antimicrobials. These bacteria could not only be a resistance reservoir but also could have potential to spread this resistance through gene transfer to pathogenic bacteria. Thus, the high prevalence of antimicrobial resistance in food animals highlights the urgent need for measures to restrict and ensure the prudent use of antimicrobials in Korea.

## 1. Introduction

*Escherichia coli* is a commensal bacterium colonizing the gastrointestinal tract of humans and animals. Most strains are harmless and seldom cause disease. However, pathogenic strains, especially enterotoxigenic *E. coli.* have been associated with food poisoning outbreaks in humans [1]. Enterotoxigenic *E. coli* was responsible for about 51,000 human deaths globally in 2016, with 24,666 in Asia, 25,075 in Africa, 796 in Latin America, and 237 in the EU [2]. In the Republic of Korea (Korea), *E. coli* was associated with 2200 annual illnesses between 2010 and 2018 [3].

*E*. *coli* strains are potential reservoirs of antimicrobial resistance genes and are considered to be excellent indicators to monitor the general level of resistance [4]. Antimicrobial resistance in commensal bacteria such as *E. coli* may serve as an early warning for the development of resistance in pathogenic bacteria [5]. A recent study in Europe has shown that more than half of the *E. coli* isolates were resistant to at least one class of antimicrobials, including those considered critically important for humans [6]. Frequent and uncontrolled use of antimicrobials in animals and humans raises the potential risk for the selection of antimicrobial resistance in commensal bacteria such as *E. coli* [7]. *E. coli* isolates with antimicrobial resistance potential can transfer from food animals to humans, either through direct contact or indirectly through the food chain [8].

Monitoring antimicrobial resistance in commensal bacteria such as *E. coli* from food animals is vital to determine the emergence of antimicrobial resistance and its associated risk to humans. Our recent study demonstrated that more than 90% of *E. coli* isolated from broiler chickens in Korea exhibited resistance to several antimicrobials, including quinolones and extended-spectrum cephalosporins [9]. Several other studies have been conducted in Korea to assess the extent of antimicrobial resistance in *E. coli* isolated from food animals [10,11,12,13,14]. However, most of these studies were on a relatively small number of isolates or isolates collected over a short duration. In addition, only a few studies have looked at the antimicrobial resistance trend. The main goal of this study is to determine the antimicrobial susceptibility and the resistance trend of *E. coli* isolated from healthy cattle, chickens, and pigs in South Korea from 2010 to 2020.

## 2. Materials and Methods

### 2.1. Isolation and Identification of E. coli

A total of 7237 *E. coli* isolates (2733 isolates from cattle, 2542 from pigs, and 1962 from chickens) were obtained from 16 laboratories/centers participating in the Korean Veterinary Antimicrobial Resistance Monitoring System (Table 1).

*E. coli* was isolated from fecal samples collected from 228 slaughterhouses during 2010–2020. The animals were delivered to the slaughterhouses from 6123 farms (≤5 samples per farm). The authors do not have information about the history of antimicrobial use in the farms, the number of animals, or the number of samples considered for this study. *E. coli* strains were isolated and identified as previously described [15]. Isolates were then confirmed by matrix-assisted laser desorption and ionization-time-of-flight mass spectrometry (MALDI-TOF, Biomerieux, Marcy L’Etoile, France). Only a single isolate per sample was considered for antimicrobial susceptibility testing.

### 2.2. Antimicrobial Susceptibility

Antimicrobial susceptibility was carried out by the broth microdilution method [1] using the commercially available Sensititre plates KRVP5F (Thermo Trek Diagnostics, Waltham, MA, USA). The isolates were tested for susceptibility toward 12 antimicrobials: amoxicillin/clavulanic acid, ampicillin, cefoxitin, ceftiofur, chloramphenicol, ciprofloxacin, colistin, gentamicin, nalidixic acid, streptomycin, tetracycline, and trimethoprim/sulfamethoxazole. *E*. *coli* ATCC 25922 and *E*. *coli* ATCC 35218 were used as quality control strains. The resulting minimum inhibitory concentration (MICs) values were interpreted according to the CLSI [16], the National Antimicrobial Resistance Monitoring System [17], and the European Committee on Antimicrobial Susceptibility Testing [18] guidelines. The MIC_50_ and MIC_90_ were calculated as the MIC that inhibited 50% and 90% of the isolates, respectively. Multi-drug resistance (MDR) was defined as resistance to three or more antimicrobial subclasses.

### 2.3. Statistical Analysis

The analysis of the antimicrobial resistance rates and Pearson correlation were conducted using Excel (Microsoft-Excel, 2016, Microsoft Corporation, Redmond, WA, USA) and Rex software (Version 3.0.3, RexSoft Inc., Seoul, Korea). *p* values less than 0.05 were considered significant.

## 3. Results

### 3.1. Antimicrobial Resistance Rate

In general, the antimicrobial resistance rate of *E. coli* isolated from chickens and pigs was significantly (*p* ≤ 0.0001) higher than that of cattle (Table 2). More than 60% of the pig and chicken isolates were resistant to ampicillin (64.1% and 72.7%), streptomycin (68.6% and 63.0%), and tetracycline (74.0% and 73.9 %, respectively). Cattle isolates presented low resistance (0.3–11.7%) to the tested antimicrobials except for streptomycin (39.2%) and tetracycline (41.4%). Antimicrobial resistance varied significantly (*p* ≤ 0.0001) among animal species. We observed, higher ciprofloxacin and nalidixic acid resistance rates in isolates from chicken (76.1% and 88.6%, respectively) than isolates from pigs (12.7% and 26.7%, respectively) and cattle (2.7% and 8.2%, respectively). In addition, significantly (*p* ≤ 0.0001) high resistance rate to chloramphenicol was observed in isolates from pigs (67.3%) than in cattle (10.2%) and chickens (45.6%). Moreover, resistance pattern screening among antimicrobials showed that a very small proportion of isolates (<5%) from cattle, chicken, and pigs demonstrated resistance against amoxicillin/clavulanic acid, cefoxitin, and colistin, while, isolates from pigs (38.6%) and chickens (42.2%) were more resistant against trimethoprim/sulfamethoxazole as compared to cattle (6.4%). Of note, we have identified 8.8%, 3.7%, and 0.7% of ceftiofur resistance in isolates from chickens, pigs, and cattle, respectively. The MIC_50_ and MIC_90_ values of the tested antimicrobials are summarized in Tables S1–S3.

### 3.2. Antimicrobial Resistance Trends

**Figure 1:**
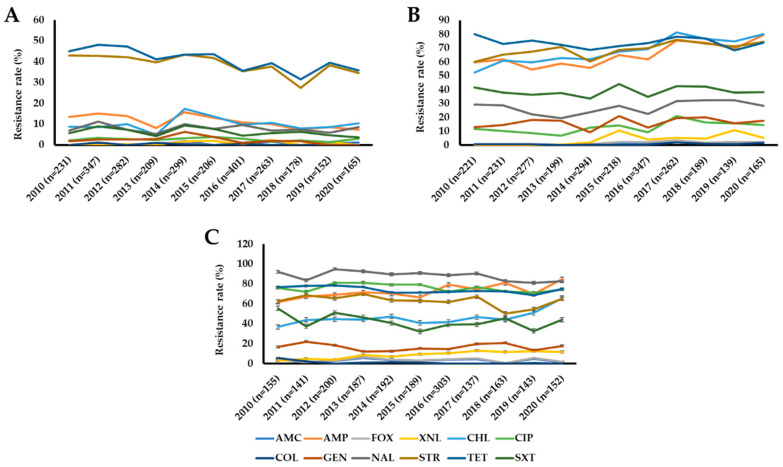
Antimicrobial resistance trends of *E*. *coli* isolates recovered from cattle (**A**), pigs (**B**), and chickens (**C**) in Korea from 2010 to 2020. Abbreviations: AMC, amoxicillin/clavulanic acid; AMP, ampicillin; CHL, chloramphenicol; CIP, ciprofloxacin; COL, colistin; FOX, cefoxitin; GEN, gentamicin; NAL, nalidixic acid; STR, streptomycin; SXT, trimethoprim/sulfamethoxazole; TET, tetracycline; XNL, ceftiofur.

The antimicrobial resistance trend varied significantly (*p* < 0.05) among isolates recovered from cattle, pigs, and chickens (Figure 1, Tables S1–S3).

The antimicrobial resistance rate screening throughout the study period showed that the isolates from cattle maintained their resistance rate below 20% against most of the tested antimicrobials except for streptomycin (27.5–43.5%) and tetracycline (31.5–48.1%) (Figure 1A, Table S1). However, we have observed a decreasing but fluctuating trend of ampicillin, streptomycin, and tetracycline resistance in cattle isolates. Pig isolates demonstrated an increasing but fluctuating resistance trend of several antimicrobials: ampicillin, amoxicillin/clavulanic acid, cefoxitin, ceftiofur, ciprofloxacin, and streptomycin (Figure 1B, Table S2). The ampicillin, tetracycline, chloramphenicol, and streptomycin resistance trend among pig isolates ranged from between 50% and 80% throughout the study period, while a moderate or low resistance rate was observed against the remaining antimicrobials. We also observed an increasing but fluctuating trend of ampicillin and ceftiofur resistance in chicken isolates, whereas colistin, nalidixic acid, and tetracycline resistance rates were decreased through time (Figure 1C, Table S3). Notably, resistance to chloramphenicol was dramatically increased in pig and chicken isolates from 2010 (52.5% and 36.8%, respectively) to 2020 (80.0% and 65.8%).

### 3.3. MDR and Antimicrobial Resistance Patterns

In the present study, 47.8% (1307/2733) of cattle isolates, 89.7% (2281/2542) of pig isolates, and 96.8% (1899/1962) of chicken isolates exhibited resistance to at least one antimicrobial agent (Table 3, Table 4 and Table 5). Overall, 56% of the isolates were multidrug-resistant. The isolates recovered from cattle showed lower MDR with 17.1% proportion as compared to isolates from chickens and pigs with a relatively higher proportion of 87.1% and 73.7%, respectively. A total of 108, 216 and 221 MDR combination patterns were observed in the cattle, pig, and chicken isolates, respectively (Tables S4–S6). Resistance to streptomycin and tetracycline was the most frequent (20.3%, 556/2733) pattern among cattle isolates (Table 3).

Resistance to ampicillin, chloramphenicol, streptomycin, and tetracycline was predominant (12.0%, 305/2542) in pig isolates (Table 4).

In addition, the major MDR pattern in chicken isolates was resistance to seven antimicrobials (10.1%, 198/1962), including ciprofloxacin and trimethoprim/sulfamethoxazole (Table 5).

## 4. Discussion

In food animals, *E. coli* strains are considered as a potential reservoir for antimicrobial resistance, and they are frequently used as a sentinel for antimicrobial resistance [19]. In this report, we studied antimicrobial resistance patterns of *E. coli* strains isolated from food animals. The isolates were collected using uniform methods of sampling and isolation. In addition, MICs of selected antimicrobials considered important in human and/or veterinary medicine were performed in a single central laboratory at the Animal and Plant Quarantine Agency, Korea. Resistance bacteria isolated from food animals may transfer to pathogenic bacteria and subsequently reduce the effectiveness of antimicrobials in humans [20]. Therefore, it is essential to know both the prevalence and trends of antimicrobial resistance in bacteria isolated from food sources. The findings of this study could be used to design and implement appropriate prevention and control strategies. [21].

Consistent with other reports in Korea [11,12,13,14] and other countries [20,22,23,24,25,26,27], we noted high levels of resistance to some specific antimicrobials in chicken and pig isolates throughout the study period. This was the case for ampicillin, streptomycin, and tetracycline, for which the occurrence of resistant strains remained continuously high (>60%). During the study period, tetracyclines, penicillin, phenicol (florfenicol), and aminoglycosides were the antibiotics most sold for use in food animals, especially pigs and chickens in Korea (APQA, 2020).

It was noteworthy that *E. coli* isolated from cattle were less frequently resistant to the tested antimicrobials compared with those isolated from chickens and pigs, a finding that has also been reported in other studies [21,28,29]. One possible reason may be that antimicrobial use is lower in cattle than in other animals. In addition, the differences in antimicrobial treatment regimens in cattle, chickens, and pigs (group vs. individual and oral (feed) vs. parenteral treatment) could contribute to the differences in antimicrobial resistance rates [30]. The slaughtering of broiler chickens at early ages (5–6 weeks), when they harbor more resistant strains than older animals [30], and continuous antimicrobial treatment until a few days before slaughter might also contribute to the occurrence of high resistant chicken isolates [21].

Overall, there was no significant difference in antimicrobial resistance in all animal species during the study period except for chloramphenicol. In 2020, in isolates from pigs and chicken, resistance to chloramphenicol increased drastically by 1.3–1.8 times when compared to 2010. Although chloramphenicol has been prohibited for use in veterinary medicine, the use of other phenicol (florfenicol) or the co-selection with unrelated antimicrobial(s) could be associated with the emergence of chloramphenicol-resistant *E. coli* [20]. In general, the high resistance to these older antimicrobials is not hard to explain because they are frequently used in food animals, especially pigs and chickens in Korea [14]. Therefore, the high-level resistance observed in chicken and pig isolates could reflect the use of these antimicrobials in poultry and pig farms.

Cephalosporins are among the critically important antimicrobial agents indicated for the treatment of MDR bacterial infections in humans [31]. In this study, the overall cefoxitin and ceftiofur resistance rates in cattle, chicken, and pig isolates remained low (<10%). Of note, we observed a trend of increasing resistance to ceftiofur or cefoxitin in chicken and pig isolates. The ceftiofur resistance rates in cattle, chicken, and pig isolates were consistent with previous reports in Korea [11], Japan [32], some European countries [24,33,34,35], and North America [33,36]. In contrast, Zhang et al. (2017) have reported a relatively high ceftiofur resistance in *E. coli* isolates from swine (16%) and broiler chickens (47%) in China [22]. Furthermore, consistent with previous studies in Korea and other countries [28,37,38,39], we found relatively high cefoxitin and ceftiofur resistance in chicken isolates than in pig and cattle isolates. Recently, we have identified extended-spectrum β-lactamase-producing (mainly CTX-M type) *Enterobacteriaceae* including *E. coli* from food animals in Korea [9,40,41]. Cefoxitin and ceftiofur-resistant strains might contribute to the emergence of resistance to other critically important cephalosporins. This could reduce the availability of these antimicrobials that can be used for critical infections [42].

Quinolones are among the priority antimicrobials used in human antimicrobial therapy [31]. Consistent with our previous study [37], we identified very high nalidixic acid and ciprofloxacin resistance in chicken isolates (88.6% and 76.1%, respectively) compared with those isolated from pigs (26.7% and 12.7%, respectively) and cattle (8.2% and 2.7%. respectively). Similarly, despite fluctuations in the levels of resistance, previous studies in Ghana [23], China [28,43], Qatar [44], and Poland [38] reported a high incidence in ciprofloxacin and/or nalidixic acid resistance more often in broiler isolates than in the pig and cattle isolates. Notably, the ciprofloxacin resistance rate in this study was higher than those reported in European countries [20] (2.0%, 0.2%, and 5.0% in cattle, pig, and chicken isolates, respectively). Ciprofloxacin is not approved for use in food animals in Korea. However, the use of other quinolones, especially enrofloxacin, in chickens might lead to an increased ciprofloxacin resistance rate. The high occurrence of ciprofloxacin resistance in healthy chicken isolates could pose a serious threat to public health.

The emergence of *E. coli* strains resistant to critically important antimicrobials such as colistin is a worldwide problem. Consistent with this study, previous reports in Korea [37] and other countries [20,21,23,28,38,45] identified a small proportion of colistin- and/or amoxicillin/clavulanic acid-resistant isolates from cattle, chickens, and pigs. In contrast, Kyung-Hyo et al. (2020) and Zhang et al. (2017) reported a relatively high occurrence of colistin (11–26%) and amoxicillin/clavulanic acid (29%) resistance in isolates from broiler chickens and/or swine in Korea and China, respectively [12,22]. Recently, the plasmid-borne *mcr-1* and *mcr-3* genes which are associated with the emergence of colistin resistance in *Enterobacteriaceae* including *E. coli* have been detected in food animals in Korea [46,47]. The World Health Organization (WHO) has classified the third and higher generation of cephalosporins, quinolones and colistin as priorities among critically important antimicrobials for treating serious infections caused by multidrug-resistant bacteria [48]. Therefore, the emergence of resistance to these antimicrobials in isolates from food animals warrants special concern and requires close monitoring.

In this study, we found high MDR rates in chicken (87.1%) and pig (73.7%) isolates compared with cattle isolates (17.1%). The prevalence of MDR remained above 60% and 80% during the whole study period for pig and chicken isolates, respectively. Similarly, several studies have reported high MDR rates in chicken (60–89%) and pig isolates (45–95%) than in cattle isolates (11–35%) [11,12,21,22,28,30]. These results suggest that stronger selective pressures for antibiotic resistance are present in the chicken and pig isolates than cattle isolates. In addition, MDR in *Enterobacteriaceae* is also complicated by the presence of mobile genetic elements, such as plasmids and transposons [49,50,51,52,53]. Our recent study demonstrated that *E. coli* isolated from healthy chickens had diverse plasmids containing mobile genetic elements and antibiotic resistance genes. Thus, the resistance noted could be owing to plasmid transfer [9].

In this study, we observed diverse MDR patterns, especially in chicken and pig isolates. The most frequent MDR patterns in cattle, chicken, and pig isolates commonly include resistance to streptomycin and tetracycline. Ciprofloxacin resistance was noted as a component of the most frequent MDR pattern in chicken isolates and should be considered highly important. Indeed, ciprofloxacin, ceftiofur, and/or colistin resistances were also noted in the less frequent MDR pattern (>7 antimicrobial agents) in chicken and pig isolates. MDR *E. coli* may spread to humans through direct contact with infected or colonized animals or their carcasses or the food chain, and poses a high risk to humans [8].

Overall, caution must be exercised when comparing and contrasting antimicrobial resistance rates and MDR profiles among studies because of the differences in the health status of animals, their history of antimicrobial use, farm management systems, and methodologies used, particularly with the determination of resistance breakpoints.

In conclusion, our study has shown a high occurrence of resistance and increasing resistance trends to commonly used antimicrobials including those considered critical for humans. Regular surveillance of antimicrobial resistance in food animals, farmworkers, and veterinarians as well as the implementation of administrative guidelines and regulations for the rational use of antimicrobials is essential to mitigate the antimicrobial resistance burden in food animals in Korea.

## Figures and Tables

**Table 1:** *E. coli* isolates obtained from feces of healthy cattle, pigs, and chickens during 2010–2020 in Korea.

	Cattle			Pigs			Chickens		
Year	No. of Slaughter-Houses	No. of Farms	No. of Isolates	No. of Slaughter-Houses	No. of Farms	No. of Isolates	No. of Slaughter- Houses	No. of Farms	No. of Isolates
2010	27	211	231	27	160	221	15	151	155
2011	29	322	347	26	195	231	14	135	141
2012	25	265	282	28	243	277	12	181	200
2013	22	207	209	28	186	199	16	183	187
2014	23	287	299	26	251	294	11	190	192
2015	23	204	206	24	204	218	13	177	189
2016	27	365	401	26	296	347	15	281	303
2017	26	260	263	28	244	262	13	133	137
2018	24	177	178	25	171	189	21	162	163
2019	27	152	152	21	136	139	22	138	143
2020	21	162	165	19	163	165	19	146	152
Total	83	2478	2733	85	2039	2542	60	1606	1962

**Table 2:** Antimicrobial resistance of *E. coli* isolated from healthy cattle, pigs, and chickens during 2010–2020 in Korea (*n* = 7237).

	% (No. of Resistant Isolates)				*p*-Value
Antimicrobials	Cattle (*n* = 2733)	Pigs (*n* = 2542)	Chickens (*n* = 1962)	Total (*n* = 7237)	
Amoxicillin/clavulanic acid	0.6 (16)	1.2 (30)	3.3 (65)	1.5 (111)	≤0.0001
Ampicillin	11.7 (320)	64.1 (1630)	72.7 (1427)	46.7 (3377)	≤0.0001
Cefoxitin	0.6 (17)	1.5 (37)	3.7 (73)	1.8 (127)	≤0.0001
Ceftiofur	0.7 (19)	3.7 (93)	8.8 (172)	3.9 (284)	≤0.0001
Chloramphenicol	10.2 (279)	67.3 (1712)	45.6 (895)	39.9 (2886)	≤0.0001
Ciprofloxacin	2.7 (75)	12.7 (322)	76.1 (1493)	26.1 (1890)	≤0.0001
Colistin	0.3 (9)	0.8 (20)	1.1 (21)	0.7 (50)	0.0021
Gentamicin	2.4 (65)	16.0 (407)	16.4 (321)	11.0 (793)	≤0.0001
Nalidixic acid	8.2 (225)	26.7 (678)	88.6 (1738)	36.5 (2641)	≤0.0001
Streptomycin	39.2 (1070)	68.6 (1743)	63.0 (1236)	55.9 (4049)	≤0.0001
Tetracycline	41.4 (1131)	74.0 (1881)	73.9 (1450)	61.7 (4462)	≤0.0001
Trimethoprim/sulfamethoxazole	6.4 (175)	38.6 (980)	42.2 (828)	27.4 (1983)	≤0.0001
MDR	17.1 (466)	73.7 (1874)	87.1 (1709)	55.9 (4049)	≤0.0001

*p* < 0.05 was considered significant change in antibiotic resistance trend. MDR, multi-drug resistant (resistant to at least three antimicrobial subclasses).

**Table 3:** Frequent resistance patterns in *E. coli* isolated from healthy cattle between 2010 and 2020 in Korea (*n* = 2733).

No. of Antimicrobials	Total No. of Isolates (%)	Most Common Pattern (No. of Isolates)
0	1426 (52.2)	-
1	233 (8.5)	TET (*n* = 122)
2	603 (22.1)	STR TET (*n* = 556)
3	219 (8.0)	NAL STR TET (*n* = 72)
4	110 (4.0)	AMP CHL STR TET (*n* = 52)
5	59 (2.2)	AMP CHL STR TET SXT (*n* = 22)
6	40 (1.5)	AMP CHL GEN STR TET SXT (*n* = 10)
		AMP CHL NAL STR TET SXT (*n* = 10)
7	25 (0.9)	AMP CHL CIP NAL STR TET SXT (*n* = 15)
8	13 (0.5)	AMP CHL CIP GEN NAL STR TET SXT (*n* = 11)
9	2 (0.1)	AMP XNL CHL CIP GEN NAL STR TET SXT (*n* = 2)
10	2 (0.1)	AMP FOX XNL CHL CIP GEN NAL STR TET SXT (*n* = 2)
11	1 (0.04)	AMC AMP FOX XNL CHL CIP GEN NAL STR TET SXT (*n* = 1)

Abbreviations: AMC, amoxicillin/clavulanic acid; AMP, ampicillin; CHL, chloramphenicol; CIP, ciprofloxacin; COL, colistin; FOX, cefoxitin; GEN, gentamicin; NAL, nalidixic acid; STR, streptomycin; SXT, trimethoprim/sulfamethoxazole; TET, tetracycline; XNL, ceftiofur).

**Table 4:** Frequent resistance patterns in *E. coli* isolated from healthy pigs between 2010 and 2020 in Korea (*n* = 2542).

No. of Antimicrobials	Total No. of Isolates (%)	Most Common Pattern (No. of Isolates)
0	261 (10.3)	
1	160 (6.3)	TET (*n* = 73)
2	232 (9.1)	STR TET (*n* = 94)
3	377 (14.8)	CHL STR TET (*n* = 92)
4	579 (22.7)	AMP CHL STR TET (*n* = 305)
5	480 (18.9)	AMP CHL STR TET SXT (*n* = 244)
6	236 (9.3)	AMP CHL NAL STR TET SXT (*n* = 63)
7	126 (5.0)	AMP CHL CIP NAL STR TET SXT (*n* = 56)
8	64 (2.5)	AMP CHL CIP GEN NAL STR TET SXT (*n* = 45)
9	20 (0.8)	AMP XNL CHL CIP GEN NAL STR TET SXT (*n* = 9)
10	5 (0.2)	AMC AMP FOX CHL CIP GEN NAL STR TET SXT (*n* = 2)
		AMP FOX XNL CHL CIP GEN NAL STR TET SXT (*n* = 2)
11	2 (0.1)	AMC AMP FOX XNL CHL CIP GEN NAL STR TET SXT (*n* = 2)

Abbreviations: AMC, amoxicillin/clavulanic acid; AMP, ampicillin; CHL, chloramphenicol; CIP, ciprofloxacin; COL, colistin; FOX, cefoxitin; GEN, gentamicin; NAL, nalidixic acid; STR, streptomycin; SXT, trimethoprim/sulfamethoxazole; TET, tetracycline; XNL, ceftiofur).

**Table 5:** Frequent resistance patterns in *E. coli* isolated from healthy chickens between 2010 and 2020 in Korea (*n* = 1962).

No. of Antimicrobials	Total No. of Isolates (%)	Most Common Pattern (No. of Isolates)
0	63 (3.2)	
1	63 (3.2)	NAL (*n* = 29)
2	123 (6.3)	CIP NAL (*n* = 55)
3	227 (11.6)	AMP CIP NAL (*n* = 47)
4	272 (13.9)	AMP CIP NAL TET (*n* = 55)
5	366 (18.7)	AMP CIP NAL STR TET (*n* = 73)
6	359 (18.3)	AMP CIP NAL STR TET SXT (*n* = 104)
7	309 (15.8)	AMP CHL CIP NAL STR TET SXT (*n* = 198)
8	142 (7.2)	AMP CHL CIP GEN NAL STR TET SXT (*n* = 107)
9	24 (1.2)	AMC AMP FOX XNL CHL CIP NAL STR TET (*n* = 7)
10	12 (0.6)	AMC AMP FOX XNL CHL CIP NAL STR TET SXT (*n* = 8)
11	2 (0.1)	AMC AMP FOX XNL CHL CIP GEN NAL STR TET SXT (*n* = 2)

Abbreviations: AMC, amoxicillin/clavulanic acid; AMP, ampicillin; CHL, chloramphenicol; CIP, ciprofloxacin; COL, colistin; FOX, cefoxitin; GEN, gentamicin; NAL, nalidixic acid; STR, streptomycin; SXT, trimethoprim/sulfamethoxazole; TET, tetracycline; XNL, ceftiofur).

## Data Availability

The data that support the findings of this study are available from the corresponding author upon reasonable request.

## Supplementary Materials

The following supporting information can be downloaded at: https://www.mdpi.com/article/10.3390/microorganisms10030524/s1, Table S1: The MIC_50_ and MIC_90_ of the tested antimicrobials against *E. coli* isolated from healthy cattle between 2010 and 2020 in Korea (*n* = 2733); Table S2. The MIC_50_ and MIC_90_ of the tested antimicrobials against *E. coli* isolated from healthy pigs between 2010 and 2020 in Korea (*n* = 2542); Table S3. The MIC_50_ and MIC_90_ of the tested antimicrobials against *E. coli* isolated from healthy chickens between 2010 and 2020 in Korea (*n* = 1962); Table S4. Antimicrobial resistance patterns of *E. coli* isolated from healthy cattle between 2010 and 2020 in Korea (*n* = 2733); Table S5. Antimicrobial resistance patterns of *E. coli* isolated from healthy pigs between 2010 and 2020 in Korea (*n* = 2542); Table S6. Antimicrobial resistance patterns of *E. coli* isolated from healthy chickens between 2010 and 2020 in Korea (*n* = 1962).
