# Supplementary material for: Antimicrobial Resistance in Escherichia coli Isolates from Healthy Food Animals in South Korea, 2010–2020
Source: Microorganisms. 2022 Feb 28;10(3):524. doi: 10.3390/microorganisms10030524 (PMC8949494; doi:10.3390/microorganisms10030524)
Supplement: Supplementary file 1 [file microorganisms-10-00524-s001.zip › microorganisms-1611122-supplementary.pdf]

Antimicrobial Resistance in *Escherichia coli* Isolates from Healthy Food Animals in South Korea, 2010–2020

Supplementary Data

Table S1. The MIC_{50} and MIC_{90} of the tested antimicrobials against *E. coli* isolated from healthy cattle between 2010 and 2020 in Korea (n=2733)

Antimicrobials	2010 (n=231)	2011 (n=347)	2012 (n=282)	2013 (n=209)	2014 (n=299)	2015 (n=206)	2016 (n=401)	2017 (n=263)	2018 (n=178)	2019 (n=152)	2020 (n=165)
**Amoxicillin/clavulanic acid**											
MIC_{50}	4	4	4	4	4	4	4	4	4	4	4
MIC_{90}	8	8	8	8	8	8	8	8	8	8	8
% Resistance (No. of isolates)	0	0.3	0 (0)	0 (0)	2.0 (6)	0 (0)	0.5 (2)	1.5 (4)	0 (0)	0.7 (1)	1.2 (2)
**Ampicillin**											
MIC_{50}	2	4	4	4	4	4	4	2	4	4	4
MIC_{90}	64	64	64	8	64	64	64	8	4	8	8
% Resistance (No. of isolates)	13.4 (31)	15.0 (52)	13.8 (39)	8.1 (17)	15.7 (47)	13.1 (27)	10.7 (43)	9.9 (26)	7.3 (13)	8.6 (13)	7.3 (12)
**Cefoxitin**											
MIC_{50}	4	4	4	4	4	4	4	4	4	4	4
MIC_{90}	8	4	8	8	8	8	8	8	8	8	8
% Resistance (No. of isolates)	0 (0)	0 (0)	0 (0)	1.0 (2)	1.0 (3)	0 (0)	1.2 (5)	2.3 (6)	0 (0)	0.7 (1)	0 (0)
**Ceftiofur**											
MIC_{50}	0.5	0.5	0.5	0.5	0.5	0.5	0.5	0.5	0.5	0.5	0.5
MIC_{90}	0.5	0.5	0.5	0.5	0.5	0.5	0.5	0.5	0.5	0.5	0.5

Resistance (%)	0 (0)	0 (0)	0.4 (1)	0 (0)	1.7 (5)	1.9 (4)	0.2 (1)	2.3 (6)	0 (0)	1.3 (2)	0 (0)
**Chloramphenicol**											
MIC_{50}	8	8	8	8	8	8	8	8	8	8	8
MIC_{90}	16	16	16	16	64	64	16	64	16	16	32
% Resistance (No. of isolates)	8.7 (20)	8.6 (30)	9.9 (28)	4.8 (10)	17.4 (52)	13.6 (28)	9.7 (39)	10.6 (28)	7.9 (14)	8.6 (13)	10.3 (17)
**Ciprofloxacin**											
MIC_{50}	0.12	0.12	0.12	0.12	0.12	0.12	0.12	0.12	0.12	0.12	0.12
MIC_{90}	0.12	0.12	0.12	0.12	0.25	0.12	0.12	0.12	0.12	0.12	0.12
% Resistance (No. of isolates)	2.2 (5)	3.5 (12)	2.8 (8)	2.4 (5)	3.3 (10)	3.9 (8)	3.0 (12)	1.5 (4)	2.2 (4)	1.3 (2)	3.0 (5)
**Colistin**											
MIC_{50}	4	4	2	2	2	2	2	2	2	2	2
MIC_{90}	4	4	2	2	2	2	2	2	2	2	2
% Resistance (No. of isolates)	0 (0)	1.2 (4)	0 (0)	1.0 (2)	0.3 (1)	0 (0)	0.5 (2)	0 (0)	0 (0)	0 (0)	0 (0)
**Gentamicin**											
MIC_{50}	1	1	1	1	1	1	1	1	1	1	1
MIC_{90}	1	1	1	1	1	1	1	1	1	1	1
% Resistance (No. of isolates)	1.7 (4)	2.6 (9)	2.5 (7)	2.9 (6)	6.4 (19)	3.9 (8)	1 (4)	1.9 (5)	1.7 (3)	0 (0)	0 (0)
**Nalidixic acid**											
MIC_{50}	2	2	2	4	2	2	2	2	2	2	2
MIC_{90}	4	64	4	4	16	4	8	4	4	4	4
% Resistance (No. of isolates)	6.9 (16)	11.2(39)	7.4 (21)	5.3 (11)	10.0(30)	7.8 (16)	9.5 (38)	6.8 (18)	7.3 (13)	5.9 (9)	8.5 (14)
**Streptomycin**											
MIC_{50}	8	8	8	8	8	8	8	16	16	16	16
MIC_{90}	128	128	128	128	128	128	128	128	128	128	128

% Resistance (No. of isolates)	42.9 (99)	42.7 (148)	42.2 (119)	39.7 (83)	43.5 (130)	41.7 (86)	35.4 (142)	37.6 (99)	27.5 (49)	38.2 (58)	34.5 (57)
**Tetracycline**											
MIC_{50}	8	8	2	2	2	2	2	2	2	2	2
MIC_{90}	128	128	128	128	128	128	128	128	128	128	128
% Resistance (No. of isolates)	45.0 (104)	48.1 (167)	47.2 (133)	41.1 (86)	43.5 (130)	43.7 (90)	35.7 (143)	39.2 (103)	31.5 (56)	39.5 (60)	35.8 (59)
**Trimethoprim/Sulfamethoxazole**											
MIC_{50}	0.12	0.12	0.12	0.12	0.12	0.12	0.12	0.12	0.12	0.12	0.12
MIC_{90}	0.25	0.5	0.5	0.5	0.5	0.5	0.25	0.25	0.25	0.25	0.25
% Resistance (No. of isolates)	5.6 (13)	8.9 (31)	7.4 (21)	4.3 (9)	9.4 (28)	7.8 (16)	4.5 (18)	5.7 (15)	6.2 (11)	4.6 (7)	3.6 (6)
MDR	16.0 (37)	18.4 (64)	17.4 (49)	12.0 (25)	23.7 (71)	18.4 (38)	17.0 (68)	15.6 (41)	11.2 (20)	17.8 (27)	16.4 (27)

MIC_{50} and MIC_{90} are the concentrations (μg/mL) at which 50% and 90% of the isolates were inhibited, respectively.

Table S2. The MIC50 and MIC90 of the tested antimicrobials against *E. coli* isolated from healthy pigs between 2010 and 2020 in Korea (n=2542)

Antimicrobials	2010 (n=221)	2011 (n=231)	2012 (n=277)	2013 (n=199)	2014 (n=294)	2015 (n=218)	2016 (n=347)	2017 (n=262)	2018 (n=189)	2019 (n=139)	2020 (n=165)
**Amoxicillin/clavulanic acid**											
MIC_{50}	4	8	8	8	8	8	8	8	8	8	8
MIC_{90}	8	8	8	8	8	8	16	16	16	16	16
% Resistance (No. of isolates)	0.5 (1)	0 (0)	0 (0)	0.5(1)	0.7(2)	1.4 (3)	2.0 (7)	2.3 (6)	1.6 (3)	2.2 (3)	2.4 (4)
**Ampicillin**											
MIC_{50}	64	64	64	64	64	64	64	64	64	64	64
MIC_{90}	64	64	64	64	64	64	64	64	64	64	64
% Resistance (No. of isolates)	59.7 (132)	61.9 (143)	54.5 (151)	58.8 (117)	55.8 (164)	65.1 (142)	62.0 (215)	75.6 (198)	74.1 (140)	69.8 (97)	79.4 (131)
**Cefoxitin**											
MIC_{50}	4	4	4	4	4	4	4	4	4	4	4
MIC_{90}	8	8	8	8	8	8	8	8	8	8	8
% Resistance (No. of isolates)	0.9 (2)	0 (0)	0 (0)	0 (0)	0.7 (2)	2.3 (5)	2.3(8)	3.4 (9)	2.1 (4)	2.2 (3)	2.4 (4)
**Ceftiofur**											
MIC_{50}	0.5	0.5	0.5	0.5	0.5	0.5	0.5	0.5	0.5	0.5	0.5
MIC_{90}	0.5	0.5	0.5	0.5	0.5	8	0.5	0.5	0.5	8	0.5
% Resistance (No. of isolates)	0.5 (1)	0.4 (1)	0 (0)	0.5 (1)	2.0 (6)	10.6(23)	4.0 (14)	5.3 (14)	4.8 (9)	10.8(15)	5.5 (9)
**Chloramphenicol**											
MIC_{50}	64	64	64	64	64	64	64	64	64	64	64
MIC_{90}	64	64	64	64	64	64	64	64	64	64	64
% Resistance (No. of isolates)	52.5 (116)	61.0 (141)	59.9 (166)	62.8 (125)	61.9 (182)	67.4 (147)	69.5 (241)	81.3 (213)	76.7 (145)	74.8 (104)	80.0 (132)
**Ciprofloxacin**											

MIC_{50}	0.12	0.12	0.12	0.12	0.12	0.12	0.12	0.12	0.12	0.25	0.12
MIC_{90}	2	1	0.5	0.5	2	2	0.5	8	8	2	8
% Resistance (No. of isolates)	11.8 (26)	10.4 (24)	8.7 (24)	7.0 (14)	12.9 (38)	14.2 (31)	9.5 (33)	21.0 (55)	16.4 (31)	15.8 (22)	14.5 (24)
**Colistin**											
MIC_{50}	4	4	2	2	2	2	2	2	2	2	2
MIC_{90}	4	4	2	2	2	2	2	2	2	2	2
% Resistance (No. of isolates)	0.9 (2)	0.9 (2)	0.7 (2)	0 (0)	0.3 (1)	0 (0)	0.6 (2)	1.9 (5)	1.1 (2)	0.7 (1)	1.8 (3)
**Gentamicin**											
MIC_{50}	1	1	1	1	1	1	1	1	1	1	1
MIC_{90}	16	16	16	16	8	32	16	32	64	32	32
% Resistance (No. of isolates)	13.1 (29)	14.7 (34)	18.4 (51)	17.6 (35)	9.5 (28)	21.1 (46)	12.7 (44)	19.5 (51)	20.1 (38)	15.8 (22)	17.6 (29)
**Nalidixic acid**											
MIC_{50}	2	2	4	4	4	4	4	4	4	4	4
MIC_{90}	128	128	128	128	128	128	128	128	128	128	128
% Resistance (No. of isolates)	29.4 (65)	28.6 (66)	22.4 (62)	19.6 (39)	23.8 (70)	28.4 (62)	22.5 (78)	31.7 (83)	32.3 (61)	32.4 (45)	28.5 (47)
**Streptomycin**											
MIC_{50}	32	64	64	64	64	64	64	64	64	64	64
MIC_{90}	128	128	128	128	128	128	128	128	128	128	128
% Resistance (No. of isolates)	60.2 (133)	65.4 (151)	67.5 (187)	70.9 (141)	60.5 (178)	68.8 (150)	70.0 (243)	76.0 (199)	73.5 (139)	71.2 (99)	74.5 (123)
**Tetracycline**											
MIC_{50}	128	64	64	64	64	64	64	64	64	64	64
MIC_{90}	128	128	128	128	128	128	128	128	128	128	128
% Resistance (No. of isolates)	80.1 (177)	73.2 (169)	75.5 (209)	72.4 (144)	68.7 (202)	71.6 (156)	73.8 (256)	78.2 (205)	77.2 (146)	68.3 (95)	73.9 (122)
**Trimethoprim/Sulfamethoxazole**											

MIC_{50}	0.25	0.25	0.25	0.25	0.25	0.25	0.25	0.5	0.25	0.25	0.25
MIC_{90}	4	4	4	4	4	4	4	4	4	4	4
% Resistance (No. of isolates)	41.6 (92)	38.1 (88)	36.5 (101)	37.7 (75)	33.7 (99)	44.0 (96)	34.9 (121)	42.7 (112)	42.3 (80)	38.1 (53)	38.2 (63)
MDR	67.4 (149)	68.8 (159)	69.7 (193)	70.4 (140)	63.6 (187)	72.9 (159)	73.8 (256)	85.5 (224)	82.0 (155)	80.6 (112)	84.8 (140)

MIC_{50} and MIC_{90} are the concentrations (μg/mL) at which 50% and 90% of the isolates were inhibited, respectively.

Table S3. The MIC_{50} and MIC_{90} of the tested antimicrobials against *E. coli* isolated from healthy chickens between 2010 and 2020 in Korea (n=1962)

Antimicrobials	2010 (n=155)	2011 (n=141)	2012 (n=200)	2013 (n=187)	2014 (n=192)	2015 (n=189)	2016 (n=303)	2017 (n=137)	2018 (n=163)	2019 (n=143)	2020 (n=152)
**Amoxicillin/clavulanic acid**											
MIC_{50}	4	8	8	8	8	8	8	8	8	8	8
MIC_{90}	8	8	8	8	8	8	16	8	8	16	16
% Resistance (No. of isolates)	3.9(6)	1.4(2)	2.5(5)	5.9(11)	3.6(7)	3.2(6)	4.0(12)	4.4(6)	0.6(1)	4.9(7)	1.3(2)
**Ampicillin**											
MIC_{50}	64	64	64	64	64	64	64	64	64	64	64
MIC_{90}	64	64	64	64	64	64	64	64	64	64	64
% Resistance (No. of isolates)	61.9(96)	67.4(95)	69.0(138)	71.7(134)	70.3(135)	66.7(126)	79.2(240)	74.5(102)	81.0(132)	69.9(100)	84.9(129)
**Cefoxitin**											
MIC_{50}	4	4	4	4	4	4	4	4	4	4	4
MIC_{90}	8	8	8	8	8	8	8	8	8	8	8
% Resistance (No. of isolates)	3.9(6)	1.4(2)	3.0(6)	6.4(12)	4.2(8)	3.2(6)	4.6(14)	5.1(7)	0.6(1)	5.6(8)	2.0(3)
**Ceftiofur**											
MIC_{50}	0.5	0.5	0.5	0.5	0.5	0.5	0.5	0.5	0.5	0.5	0.5
MIC_{90}	0.5	0.5	0.5	1	1	4	8	8	8	8	8
% Resistance (No. of isolates)	3.2(5)	5.0(7)	4.0(8)	8.6(16)	6.8(13)	9.5(18)	10.6(32)	13.1(18)	11.7(19)	12.6(18)	11.8(18)
**Chloramphenicol**											
MIC_{50}	8	8	8	8	8	8	8	16	8	32	64
MIC_{90}	64	64	64	64	64	64	64	64	64	64	64
% Resistance (No. of isolates)	36.8(57)	44.0(62)	44.5(89)	44.4(83)	47.4(91)	40.7(77)	41.9(127)	46.7(64)	44.2(72)	51.0(73)	65.8(100)
**Ciprofloxacin**											

MIC$_{50}$	8	8	8	8	8	8	8	8	8	8	8
MIC$_{90}$	16	16	16	16	16	16	16	16	16	16	16
% Resistance (No. of isolates)	76.1(118)	72.3(102)	81.0(162)	81.3(152)	79.2(152)	79.4(150)	72.3(219)	76.6(105)	72.4(118)	71.3(102)	74.3(113)
**Colistin**											
MIC$_{50}$	4	4	2	2	2	2	2	2	2	2	2
MIC$_{90}$	4	4	2	2	2	2	2	2	2	2	2
% Resistance (No. of isolates)	5.8(9)	2.1(3)	0.0 (0)	1.1(2)	1.6(3)	1.1(2)	0.3(1)	0.0 (0)	0.0 (0)	0.7(1)	0.0 (0)
**Gentamicin**											
MIC$_{50}$	1	1	1	1	1	1	1	1	1	1	1
MIC$_{90}$	32	64	64	32	32	64	64	64	64	64	64
% Resistance (No. of isolates)	16.8(26)	22.0(31)	18.5(37)	12.3(23)	12.5(24)	15.3(29)	14.5(44)	19.7(27)	20.9(34)	13.3(19)	17.8(27)
**Nalidixic acid**											
MIC$_{50}$	128	128	128	128	128	128	128	128	128	128	128
MIC$_{90}$	128	128	128	128	128	128	128	128	128	128	128
% Resistance (No. of isolates)	92.3(143)	83.7(118)	95.0(190)	92.5(173)	89.6(172)	91.0(172)	88.8(269)	90.5(124)	82.8(135)	81.1(116)	82.9(126)
**Streptomycin**											
MIC$_{50}$	64	128	128	128	64	64	64	64	32	64	64
MIC$_{90}$	128	128	128	128	128	128	128	128	128	128	128
% Resistance (No. of isolates)	62.6(97)	68.8(97)	65.5(131)	70.1(131)	63.5(122)	63.0(119)	62.0(188)	67.2(92)	50.3(82)	54.5(78)	65.1(99)
**Tetracycline**											
MIC$_{50}$	64	64	64	64	64	64	64	64	64	64	64
MIC$_{90}$	128	128	128	128	128	128	128	128	128	128	128
% Resistance (No. of isolates)	76.8(119)	78.0(110)	78.5(157)	77.0(144)	71.4(137)	71.4(135)	71.9(218)	73.0(100)	72.4(118)	68.5(98)	75.0(114)

**Trimethoprim/Sulfamethoxazole**											
MIC_{50}	4	1	4	0.5	0.5	0.25	0.25	0.25	0.5	0.12	0.25
MIC_{90}	4	4	4	4	4	4	4	4	4	4	4
% Resistance (No. of isolates)	55.5(86)	37.6(53)	51.0(102)	46.5(87)	40.6(78)	32.3(61)	39.3(119)	39.4(54)	45.4(74)	32.9(47)	44.1(67)
MDR	86.5 (134)	86.5 (122)	91.0 (182)	88.8 (166)	89.1 (171)	83.6 (158)	86.8 (263)	86.1 (118)	85.3 (139)	84.6 (121)	88.8 (135)

MIC_{50} and MIC_{90} are the concentrations (μg/mL) at which 50% and 90% of the isolates were inhibited, respectively.

Table S4. Antimicrobial resistance patterns of *E. coli* isolated from healthy cattle between 2010 and 2020 in Korea (n=2733).

No. of antimicrobials (No of isolates)	Resistance patterns (No. of isolates)
0 (n=1426)	-
1 (n=233)	TET (n=122), STR (n=69), NAL (n=22), AMP (n=10), CHL (n=7), COL (n=2), FOX (n=1)
2 (n=603)	STR TET (n=556), AMP STR (n=14), NAL TET (n=6), TET SXT (n=6), AMP TET (n=5), CHL TET (n=4), NAL STR (n=3), AMP CHL (n=2), CHL STR (n=2), AMC AMP (n=1), AMP XNL (n=1), CIP NAL (n=1), GEN STR (n=1), STR SXT (n=1)
3 (n=219)	NAL STR TET (n=72), AMP STR TET (n=58), CHL STR TET (n=43), AMP CHL STR (n=8), AMP TET SXT (n=7), GEN STR TET (n=5), AMP CHL TET (n=4), AMP STR SXT (n=3), CIP NAL TET (n=3), COL STR TET (n=3), STR TET SXT (n=3), CHL GEN NAL (n=2), CHL NAL TET (n=2), AMC AMP FOX (n=1), AMP CIP NAL (n=1), AMP NAL TET (n=1), CHL TET SXT (n=1), CIP NAL SXT (n=1), FOX STR TET (n=1)
4 (n=110)	AMP CHL STR TET (n=52), CHL STR TET SXT (n=14), AMP STR TET SXT (n=6), AMP NAL STR TET (n=4), CHL NAL STR TET (n=4), AMP CHL STR SXT (n=3), AMP CHL TET SXT (n=3), CIP NAL STR TET (n=3), NAL STR TET SXT (n=3), AMC AMP FOX XNL (n=2), AMC AMP STR TET (n=2), AMP CIP NAL TET (n=2), AMP NAL STR SXT (n=2), CHL CIP NAL STR (n=2), CIP NAL STR SXT (n=2), AMP CHL GEN TET (n=1), AMP NAL TET SXT (n=1), AMP XNL TET SXT (n=1), CHL GEN STR TET (n=1), FOX CHL STR TET (n=1), GEN STR TET SXT (n=1)
5 (n=59)	AMP CHL STR TET SXT (n=22), CHL GEN STR TET SXT (n=6), AMP CHL NAL STR TET (n=5), AMC AMP CHL STR TET (n=4), CHL NAL STR TET SXT (n=4), AMP CHL CIP NAL STR (n=2), AMP CHL GEN STR TET (n=2), AMP CHL GEN TET SXT (n=2), AMP CHL NAL TET SXT (n=2), AMP GEN STR TET SXT (n=2), AMP NAL STR TET SXT (n=2), AMC AMP FOX NAL TET (n=1), AMC AMP STR TET SXT (n=1), AMP CHL NAL STR SXT (n=1), AMP CIP NAL STR TET (n=1), CHL CIP NAL STR TET (n=1), CIP NAL STR TET SXT (n=1)
6 (n=40)	AMP CHL GEN STR TET SXT (n=10), AMP CHL NAL STR TET SXT (n=10), AMP CHL CIP NAL STR SXT (n=3), AMP CIP NAL STR TET SXT (n=3), AMP CHL CIP NAL TET SXT (n=2), CHL CIP NAL STR TET SXT (n=2), AMC AMP FOX CHL CIP NAL (n=1), AMP CHL CIP NAL STR TET (n=1), AMP FOX XNL CHL STR TET (n=1), AMP FOX XNL GEN STR TET (n=1), AMP GEN NAL STR TET SXT (n=1), AMP XNL CHL CIP NAL TET (n=1), AMP XNL CHL GEN TET SXT (n=1), AMP XNL NAL STR TET SXT (n=1), CHL CIP GEN NAL STR TET (n=1), CIP GEN NAL STR TET SXT (n=1), CIP NAL STR TET SXT (n=1)
7 (n=25)	AMP CHL CIP NAL STR TET SXT (n=15), AMP CHL GEN NAL STR TET SXT (n=2), AMP CIP GEN NAL STR TET SXT (n=2), AMP XNL CHL CIP GEN NAL SXT (n=2), AMC AMP FOX XNL CIP GEN TET (n=1), AMC AMP FOX XNL GEN STR TET (n=1), AMP CHL CIP GEN NAL STR SXT (n=1), AMP CHL CIP GEN NAL TET SXT (n=1)
8 (n=13)	AMP CHL CIP GEN NAL STR TET SXT (n=11), AMP FOX CHL CIP NAL STR TET SXT (n=1), AMP FOX XNL CHL CIP GEN NAL STR (n=1)
9 (n=2)	AMP XNL CHL CIP GEN NAL STR TET SXT (n=2)
10 (n=2)	AMP FOX XNL CHL CIP GEN NAL STR TET SXT (n=2)
11 (n=1)	AMC AMP FOX XNL CHL CIP GEN NAL STR TET SXT (n=1)

Abbreviations: AMC, amoxicillin/clavulanic acid; AMP, ampicillin; CHL, chloramphenicol; CIP, ciprofloxacin; COL, colistin; FOX, cefoxitin; GEN, gentamicin; NAL, nalidixic acid; STR, streptomycin; SXT, trimethoprim/sulfamethoxazole; TET, tetracycline; XNL, ceftiofur)

Table S5. Antimicrobial resistance patterns of *E. coli* isolated from healthy pigs between 2010 and 2020 in Korea (n=2542)

No. of antimicrobials (No of isolates)	Resistance patterns (No. of isolates)
0 (n=261)	
1 (n=160)	TET (n=73), NAL (n=24), STR (n=22), CHL (n=19), AMP (n=17), SXT (n=3), COL (n=1), GEN (n=1)
2 (n=232)	STR TET (n=94), CHL TET (n=27), AMP STR (n=19), AMP TET (n=19), AMP CHL (n=15), CHL STR (n=15), NAL TET (n=8), CHL SXT (n=7), CIP NAL (n=6), GEN STR (n=4), STR SXT (n=4), CHL NAL (n=3), TET SXT (n=3), AMP SXT (n=2), NAL STR (n=2), AMC AMP (n=1), AMP NAL (n=1), AMP XNL (n=1), CHL GEN (n=1)
3 (n=377)	CHL STR TET (n=92), AMP CHL TET (n=72), AMP STR TET (n=57), AMP CHL STR (n=35), CHL TET SXT (n=13), AMP STR SXT (n=10), CHL STR SXT (n=10), NAL STR TET (n=10), AMP NAL STR (n=9), AMP CHL SXT (n=8), AMP TET SXT (n=8), CHL GEN STR (n=8), STR TET SXT (n=8), GEN STR TET (n=6), CHL CIP NAL (n=4), CHL NAL TET (n=4), AMP GEN TET (n=3), AMP NAL SXT (n=3), AMP CHL GEN (n=2), AMP CIP NAL (n=2), CIP NAL STR (n=2), AMP CHL NAL (n=1), AMP GEN SXT (n=1), AMP XNL STR (n=1), CHL GEN NAL (n=1), CHL NAL STR (n=1), CHL NAL SXT (n=1), CIP NAL SXT (n=1), CIP NAL TET (n=1), GEN STR SXT (n=1), NAL STR SXT (n=1), NAL TET SXT (n=1)
4 (n=579)	AMP CHL STR TET (n=305), AMP CHL TET SXT (n=69), AMP STR TET SXT (n=35), CHL STR TET SXT (n=28), CHL GEN STR TET (n=22), AMP CHL STR SXT (n=20), CHL NAL STR TET (n=17), AMP NAL STR TET (n=9), AMP CHL NAL TET (n=8), AMP GEN STR TET (n=7), AMP GEN STR SXT (n=6), AMP CHL GEN STR (n=5), AMP CHL NAL SXT (n=5), CHL CIP NAL TET (n=4), NAL STR TET SXT (n=4), AMP CHL CIP NAL (n=3), AMP CHL NAL STR (n=3), AMP CIP NAL STR (n=3), AMP NAL STR SXT (n=3), CHL GEN STR SXT (n=3), AMP CHL GEN SXT (n=2), AMP CIP NAL TET (n=2), AMP XNL CIP NAL (n=2), CHL GEN NAL TET (n=2), CHL NAL TET SXT (n=2), CIP NAL STR TET (n=2), AMP CHL GEN TET (n=1), AMP NAL TET SXT (n=1), AMP XNL CHL GEN (n=1), AMP XNL NAL TET (n=1), AMP XNL STR TET (n=1), CHL GEN TET SXT (n=1), CHL NAL STR SXT (n=1), GEN NAL STR TET (n=1)
5 (n=480)	AMP CHL STR TET SXT (n=244), AMP CHL NAL STR TET (n=49), AMP CHL GEN STR TET (n=30), CHL GEN STR TET SXT (n=15), CHL NAL STR TET SXT (n=13), AMP CHL NAL TET SXT (n=10), AMP CHL CIP NAL STR (n=9), AMP CHL GEN STR SXT (n=9), CHL CIP NAL STR TET (n=9), AMP CHL GEN TET SXT (n=8), AMP NAL STR TET SXT (n=8), AMP GEN STR TET SXT (n=7), CHL GEN NAL STR TET (n=7), GEN NAL STR TET SXT (n=7), AMP CHL CIP NAL TET (n=6), CHL CIP NAL TET SXT (n=5), AMP XNL CHL NAL STR (n=4), AMP CHL GEN NAL STR (n=3), AMP CIP NAL TET SXT (n=3), AMP XNL CHL STR TET (n=3), CHL CIP GEN NAL SXT (n=3), AMC AMP CHL STR TET (n=2), AMP CHL CIP NAL SXT (n=2), AMP CHL CIP STR TET (n=2), AMP CHL NAL STR SXT (n=2), AMP CIP NAL STR TET (n=2), AMP GEN NAL STR SXT (n=2), AMP XNL CHL TET SXT (n=2), AMC AMP FOX CIP NAL (n=1), AMP CHL CIP GEN NAL (n=1), AMP CHL CIP TET SXT (n=1), AMP CHL COL STR SXT (n=1), AMP CHL COL STR TET (n=1),

	AMP CHL GEN NAL TET (n=1), AMP CIP GEN NAL STR (n=1), AMP GEN NAL STR TET (n=1), AMP XNL CHL CIP NAL (n=1), AMP XNL CHL NAL TET (n=1), AMP XNL CHL STR SXT (n=1), CHL CIP GEN NAL TET (n=1), CHL COL GEN STR TET (n=1), CIP NAL STR TET SXT (n=1)
6 (n=236)	AMP CHL NAL STR TET SXT (n=63), AMP CHL GEN STR TET SXT (n=49), AMP CHL CIP NAL STR TET (n=17), AMP CHL GEN NAL STR TET (n=12), AMP XNL CHL STR TET SXT (n=12), AMP GEN NAL STR TET SXT (n=10), AMP CIP NAL STR TET SXT (n=8), AMP CHL CIP NAL TET SXT (n=7), AMP CHL GEN NAL TET SXT (n=7), CHL CIP NAL STR TET SXT (n=7), AMP CHL CIP NAL STR SXT (n=6), AMP CHL CIP GEN NAL STR (n=4), AMC AMP FOX XNL STR TET (n=3), AMP XNL CHL CIP NAL TET (n=3), AMP CHL CIP GEN NAL SXT (n=2), AMP CHL CIP STR TET SXT (n=2), AMP CIP GEN NAL STR TET (n=2), CHL CIP GEN NAL STR SXT (n=2), CHL CIP GEN NAL STR TET (n=2), CHL GEN NAL STR TET SXT (n=2), AMC AMP FOX XNL CHL TET (n=1), AMP CHL CIP COL STR SXT (n=1), AMP CHL CIP GEN NAL TET (n=1), AMP CHL COL GEN STR TET (n=1), AMP CHL COL STR TET SXT (n=1), AMP CHL GEN NAL STR SXT (n=1), AMP FOX CHL CIP NAL TET (n=1), AMP FOX CHL STR TET SXT (n=1), AMP XNL CHL COL NAL STR (n=1), AMP XNL CHL GEN NAL STR (n=1), AMP XNL CHL NAL STR SXT (n=1), AMP XNL CHL NAL STR TET (n=1), AMP XNL CHL NAL TET SXT (n=1), AMP XNL CIP NAL STR SXT (n=1), AMP XNL CIP NAL STR TET (n=1), CHL CIP GEN NAL TET SXT (n=1)
7 (n=126)	AMP CHL CIP NAL STR TET SXT (n=56), AMP CHL CIP GEN NAL STR TET (n=19), AMP CHL GEN NAL STR TET SXT (n=16), AMP CHL CIP GEN NAL STR SXT (n=7), CHL CIP GEN NAL STR TET SXT (n=4), AMC AMP FOX XNL CHL STR TET (n=2), AMP CHL COL GEN STR TET SXT (n=2), AMP CIP GEN NAL STR TET SXT (n=2), AMP XNL CHL CIP NAL TET SXT (n=2), AMP XNL CHL GEN STR TET SXT (n=2), AMP XNL CHL NAL STR TET SXT (n=2), AMC AMP FOX XNL CHL STR SXT (n=1), AMC AMP FOX XNL STR TET SXT (n=1), AMP CHL CIP COL NAL STR TET (n=1), AMP CHL CIP GEN NAL TET SXT (n=1), AMP CHL COL NAL STR TET SXT (n=1), AMP FOX CHL CIP STR TET SXT (n=1), AMP FOX CHL GEN STR TET SXT (n=1), AMP XNL CHL CIP NAL STR SXT (n=1), AMP XNL CHL CIP NAL STR TET (n=1), AMP XNL CHL GEN NAL STR SXT (n=1), AMP XNL CHL GEN NAL TET SXT (n=1), CHL CIP COL GEN NAL TET SXT (n=1)
8 (n=64)	AMP CHL CIP GEN NAL STR TET SXT (n=45), AMP CHL CIP COL NAL STR TET SXT (n=3), AMP XNL CHL CIP GEN NAL STR SXT (n=3), AMP XNL CHL CIP GEN NAL STR TET (n=3), AMC AMP FOX XNL CHL GEN STR TET (n=2), AMC AMP FOX XNL CHL NAL STR TET (n=2), AMC AMP FOX XNL CHL STR TET SXT (n=2), AMP XNL CHL CIP NAL STR TET SXT (n=2), AMP FOX CHL CIP NAL STR TET SXT (n=1), AMP FOX XNL CHL NAL STR TET SXT (n=1)
9 (n=20)	AMP XNL CHL CIP GEN NAL STR TET SXT (n=9), AMC AMP FOX XNL CHL NAL STR TET SXT (n=3), AMC AMP FOX XNL CHL GEN STR TET SXT (n=2), AMP CHL CIP COL GEN NAL STR TET SXT (n=2), AMC AMP FOX CHL CIP GEN NAL TET SXT (n=1), AMC AMP FOX CIP GEN NAL STR TET SXT (n=1), AMP FOX CHL CIP GEN NAL STR TET SXT (n=1), FOX CHL CIP COL GEN NAL STR TET SXT (n=1)
10 (n=5)	AMC AMP FOX CHL CIP GEN NAL STR TET SXT (n=2), AMP FOX XNL CHL CIP GEN NAL STR TET SXT (n=2), AMC AMP FOX XNL CHL CIP GEN NAL STR TET (n=1)
11 (n=2)	AMC AMP FOX XNL CHL CIP GEN NAL STR TET SXT (n=2)

Abbreviations: AMC, amoxicillin/clavulanic acid; AMP, ampicillin; CHL, chloramphenicol; CIP, ciprofloxacin; COL, colistin; FOX, cefoxitin; GEN, gentamicin; NAL, nalidixic acid; STR, streptomycin; SXT, trimethoprim/sulfamethoxazole; TET, tetracycline; XNL, ceftiofur)

Table S6. Antimicrobial resistance patterns of E. *coli* isolated from healthy chickens between 2010 and 2020 in Korea (n=1962)

No. of antimicrobials (No of isolates)	Resistance patterns (No. of isolates)
0 (n=63)	-
1 (n=63)	NAL (n=29), AMP (n=16), TET (n=10), STR (n=5), CHL (n=1), CIP (n=1), GEN (n=1)
2 (n=123)	CIP NAL (n=55), AMP TET (n=20), NAL STR (n=15), NAL TET (n=15), AMP NAL (n=4), STR TET (n=4), AMP CHL (n=2), AMP STR (n=2), NAL SXT (n=2), AMP GEN (n=1), AMP XNL (n=1), CHL NAL (n=1), STR SXT (n=1)
3 (n=227)	AMP CIP NAL (n=47), CIP NAL STR (n=46), CIP NAL TET (n=41), AMP NAL TET (n=14), AMP CHL TET (n=11), AMP STR TET (n=11), NAL STR TET (n=9), NAL TET SXT (n=7), CHL CIP NAL (n=5), AMP CIP TET (n=4), CIP NAL SXT (n=4), AMP STR SXT (n=3), AMP TET SXT (n=3), GEN STR SXT (n=3), NAL STR SXT (n=3), AMP CHL NAL (n=2), AMP NAL STR (n=2), CHL NAL TET (n=2), AMP GEN STR (n=1), AMP GEN TET (n=1), AMP NAL SXT (n=1), AMP XNL CHL (n=1), AMP XNL NAL (n=1), CHL NAL STR (n=1), CHL STR TET (n=1), CIP GEN NAL (n=1), COL STR TET (n=1), GEN NAL TET (n=1)
4 (n=272)	AMP CIP NAL TET (n=55), CIP NAL STR TET (n=36), AMP CIP NAL STR (n=25), CIP NAL TET SXT (n=21), AMP CHL STR TET (n=16), AMP NAL STR TET (n=13), AMP CHL CIP NAL (n=12), CHL CIP NAL STR (n=11), AMP CHL NAL TET (n=9), AMP STR TET SXT (n=9), CHL NAL STR TET (n=8), AMP NAL TET SXT (n=5), AMP XNL CIP NAL (n=5), AMP CIP GEN NAL (n=4), AMP NAL STR SXT (n=4), CHL CIP NAL TET (n=4), CIP NAL STR SXT (n=4), AMC AMP FOX XNL (n=3), NAL STR TET SXT (n=3), AMP FOX CIP NAL (n=2), CHL GEN STR TET (n=2), CHL NAL TET SXT (n=2), CHL STR TET SXT (n=2), CIP GEN NAL STR (n=2), CIP GEN NAL TET (n=2), GEN NAL STR TET (n=2), AMP CHL GEN NAL (n=1), AMP CHL NAL STR (n=1), AMP CHL STR SXT (n=1), AMP CIP COL NAL (n=1), AMP CIP NAL SXT (n=1), AMP GEN NAL STR (n=1), AMP GEN NAL TET (n=1), AMP GEN STR TET (n=1), AMP XNL NAL TET (n=1), CHL CIP NAL SXT (n=1), FOX CHL CIP NAL (n=1)
5 (n=366)	AMP CIP NAL STR TET (n=73), AMP CHL CIP NAL TET (n=58), CHL CIP NAL STR TET (n=44), AMP CIP NAL TET SXT (n=20), AMP NAL STR TET SXT (n=20), CIP NAL STR TET SXT (n=19), AMP CIP NAL STR SXT (n=18), AMP CHL NAL STR TET (n=13), AMP CHL STR TET SXT (n=12), AMP XNL CIP NAL TET (n=12), AMP CHL CIP NAL SXT (n=7), AMP CHL CIP NAL STR (n=6), AMP CIP GEN NAL STR (n=6), AMP CIP GEN NAL TET (n=5), AMP CHL NAL TET SXT (n=4), AMP GEN NAL TET SXT (n=4), AMP XNL CHL CIP NAL (n=4), AMC AMP FOX XNL NAL (n=3), AMP CHL CIP GEN NAL (n=3), AMP CIP GEN NAL SXT (n=3), AMP XNL CIP NAL STR (n=3), CIP GEN NAL STR TET (n=3), AMC AMP FOX XNL TET (n=2), AMP XNL CHL STR TET (n=2), AMP XNL NAL STR TET (n=2), CHL CIP GEN NAL STR (n=2), CHL CIP NAL TET SXT (n=2), CIP GEN NAL STR

	SXT (n=2), AMC AMP FOX CIP NAL (n=1), AMC AMP FOX NAL TET (n=1), AMP CHL CIP COL NAL (n=1), AMP CHL GEN NAL TET (n=1), AMP CHL GEN STR TET (n=1), AMP CHL NAL STR SXT (n=1), AMP FOX GEN NAL TET (n=1), AMP XNL CHL NAL STR (n=1), AMP XNL CHL NAL TET (n=1), AMP XNL CIP GEN NAL (n=1), AMP XNL GEN STR TET (n=1), AMP XNL STR TET SXT (n=1), CHL CIP NAL STR SXT (n=1), CHL NAL STR TET SXT (n=1)
6 (n=359)	AMP CIP NAL STR TET SXT (n=104), AMP CHL CIP NAL STR TET (n=99), AMP CHL CIP NAL TET SXT (n=39), CHL CIP NAL STR TET SXT (n=17), AMP CHL CIP NAL STR SXT (n=11), AMP CIP GEN NAL STR TET (n=10), AMP CHL NAL STR TET SXT (n=9), AMP CHL GEN NAL STR TET (n=6), AMC AMP FOX XNL CIP NAL (n=5), AMP CHL CIP GEN NAL TET (n=4), AMP CIP GEN NAL STR SXT (n=4), AMP CIP GEN NAL TET SXT (n=4), AMP XNL CHL CIP NAL SXT (n=3), AMP XNL CHL CIP NAL TET (n=3), AMP XNL CHL NAL STR TET (n=3), AMP XNL CIP NAL STR TET (n=3), AMP XNL GEN NAL STR TET (n=3), CHL CIP GEN NAL STR TET (n=3), AMP CHL CIP GEN NAL STR (n=2), AMP CHL CIP GEN NAL SXT (n=2), AMP XNL CIP GEN NAL STR (n=2), AMP XNL CIP GEN NAL TET (n=2), AMP XNL CIP NAL STR SXT (n=2), AMC AMP FOX XNL GEN STR (n=1), AMC AMP FOX XNL NAL STR (n=1), AMC AMP FOX XNL NAL TET (n=1), AMP CHL CIP COL NAL TET (n=1), AMP CHL GEN NAL TET SXT (n=1), AMP CHL GEN STR TET SXT (n=1), AMP CIP COL NAL STR SXT (n=1), AMP CIP COL NAL STR TET (n=1), AMP CIP COL NAL TET SXT (n=1), AMP XNL CHL CIP GEN NAL (n=1), AMP XNL CHL CIP NAL STR (n=1), AMP XNL CHL STR TET SXT (n=1), AMP XNL CIP NAL TET SXT (n=1), AMP XNL NAL STR TET SXT (n=1), CHL CIP COL GEN NAL TET (n=1), CHL CIP GEN NAL STR SXT (n=1), CHL CIP GEN NAL TET SXT (n=1), CHL GEN NAL STR TET SXT (n=1), CIP GEN NAL STR TET SXT (n=1)
7 (n=309)	AMP CHL CIP NAL STR TET SXT (n=198), AMP CIP GEN NAL STR TET SXT (n=32), AMP CHL CIP GEN NAL STR TET (n=24), AMP XNL CHL CIP NAL STR TET (n=8), CHL CIP GEN NAL STR TET SXT (n=6), AMP CHL GEN NAL STR TET SXT (n=5), AMP CHL CIP GEN NAL TET SXT (n=4), AMP XNL CHL CIP NAL TET SXT (n=4), AMP CHL CIP GEN NAL STR SXT (n=3), AMP XNL CIP GEN NAL STR TET (n=3), AMP XNL CIP NAL STR TET SXT (n=3), AMC AMP FOX XNL CIP NAL STR (n=2), AMC AMP FOX XNL CIP NAL TET (n=2), AMP XNL CHL CIP GEN NAL SXT (n=2), AMP XNL CHL CIP GEN NAL TET (n=2), AMC AMP FOX XNL CIP NAL SXT (n=1), AMC AMP FOX XNL NAL STR SXT (n=1), AMC AMP FOX XNL NAL STR TET (n=1), AMP CHL CIP COL NAL STR TET (n=1), AMP CHL CIP COL NAL TET SXT (n=1), AMP CIP COL NAL STR TET SXT (n=1), AMP FOX CHL CIP NAL STR SXT (n=1), AMP FOX CHL NAL STR TET SXT (n=1), AMP XNL CHL CIP GEN NAL STR (n=1), AMP XNL CHL GEN NAL STR TET (n=1), AMP XNL CIP GEN NAL TET SXT (n=1)

8 (n=142)	AMP CHL CIP GEN NAL STR TET SXT (n=107), AMP XNL CHL CIP NAL STR TET SXT (n=13), AMC AMP FOX XNL CIP NAL STR TET (n=4), AMC AMP FOX XNL CIP NAL TET SXT (n=4), AMC AMP FOX CHL CIP NAL STR TET (n=3), AMP XNL CIP GEN NAL STR TET SXT (n=3), AMP XNL CHL CIP GEN NAL TET SXT (n=2), AMC AMP FOX XNL NAL STR TET SXT (n=1), AMP CHL CIP COL NAL STR TET SXT (n=1), AMP CIP COL GEN NAL STR TET SXT (n=1), AMP FOX CHL CIP NAL STR TET SXT (n=1), AMP XNL CHL CIP COL NAL STR TET (n=1), AMP XNL CHL CIP GEN NAL STR TET (n=1)
9 (n=24)	AMC AMP FOX XNL CHL CIP NAL STR TET (n=7), AMC AMP FOX XNL CIP NAL STR TET SXT (n=5), AMP XNL CHL CIP GEN NAL STR TET SXT (n=5), AMP CHL CIP COL GEN NAL STR TET SXT (n=3), AMC AMP FOX CHL CIP NAL STR TET SXT (n=1), AMC AMP FOX XNL COL NAL STR TET SXT (n=1), AMP FOX CHL CIP GEN NAL STR TET SXT (n=1), AMP XNL CHL CIP COL NAL STR TET SXT (n=1)
10 (n=12)	AMC AMP FOX XNL CHL CIP NAL STR TET SXT (n=8), AMC AMP FOX XNL CIP GEN NAL STR TET SXT (n=2), AMC AMP FOX CHL CIP GEN NAL STR TET SXT (n=1), AMC AMP FOX XNL CHL CIP GEN NAL STR TET (n=1)
11 (n=2)	AMC AMP FOX XNL CHL CIP GEN NAL STR TET SXT (n=2)

Abbreviations: AMC, amoxicillin/clavulanic acid; AMP, ampicillin; CHL, chloramphenicol; CIP, ciprofloxacin; COL, colistin; FOX, cefoxitin; GEN, gentamicin; NAL, nalidixic acid; STR, streptomycin; SXT, trimethoprim/sulfamethoxazole; TET, tetracycline; XNL, ceftiofur)
